# Genome-Wide Identification and Expression Analysis of the G-Protein Gene Family in Barley Under Abiotic Stresses

**DOI:** 10.3390/plants13243521

**Published:** 2024-12-17

**Authors:** Ailing Han, Zhengyuan Xu, Zhenyu Cai, Yuling Zheng, Mingjiong Chen, Liyuan Wu, Qiufang Shen

**Affiliations:** 1 Institute of Crop Science, College of Agriculture and Biotechnology, Zhejiang University, Hangzhou 310058, China; 22216196@zju.edu.cn (A.H.); 11816026@zju.edu.cn (Z.X.); zhenyucai@zju.edu.cn (Z.C.); 22316030@zju.edu.cn (Y.Z.); 22316013@zju.edu.cn (M.C.); 2 Zhongyuan Institute, Zhejiang University, Zhengzhou 450000, China; 3 Department of Architectural Engineering, Shaoxing University Yuanpei College, Shaoxing 312000, China

**Keywords:** barley, G-protein gene family, abiotic stresses, expression analysis, salt stress

## Abstract

Heterotrimeric G-proteins are fundamental signal transducers highly conserved in plant species, which play crucial roles in regulating plant growth, development, and responses to abiotic stresses. Identification of G-protein members and their expression patterns in plants are essential for improving crop resilience against environmental stresses. Here, we identified eight heterotrimeric G-protein genes localized on four chromosomes within the barley genome by using comprehensive genome-wide analysis. Phylogenetic analysis classified these genes into four distinct subgroups with obvious evolutionary relationships. Further analysis on gene structure, protein motif, and structure indicated that G-proteins within each evolutionary branch exhibited similar exon-intron organization, conserved motif patterns, and structural features. Collinearity analysis showed no significant collinear relationships among those G-protein genes, indicating a unique evolutionary trajectory within barley. Moreover, *cis*-regulatory elements detected in the upstream sequences of these genes were involved in response to plant hormones and signaling molecules. Expression analyses revealed tissue-specific expression patterns and differential regulation in response to abiotic stresses. The expression patterns of G-protein genes were further validated using a quantitative real-time PCR (qRT-PCR) technique, indicating the reliability of transcriptomic data, as well as special responses to salt, drought, and waterlogging stresses. These findings may provide underlying mechanisms by which G-protein genes participate in salt tolerance of barley, and also highlight candidate genes for potential genetic engineering applications in improving crop resilience to salinity stress.

## 1. Introduction

The challenge of climate change and global population brings an urgent threat to food security, exacerbating pressure on crop production systems worldwide [1]. With the rise of global temperatures and extreme weather events, the resilience of crops to abiotic stresses such as drought, salinity, and waterlogging stresses is crucial for sustaining agricultural productivity [2,3]. However, there is still a lack of knowledge underlying molecular mechanisms driving stress tolerance in plant responses [4]. Understanding these mechanisms is key to unlocking genetic modifications that enhances plant resilience and productivity under adverse conditions [4].

Heterotrimeric G-proteins, ubiquitous in eukaryotes, are pivotal signal transducers in a wide array of biological processes, mediating responses to extracellular stimuli in both plants and animals [5,6]. Structurally, G-proteins consist of three subunits: Gα, Gβ, and Gγ, with extra-large Gα subunits (XLGs) sharing homology with Gα [7]. The diversity of G-protein subunits is quite extensive in humans with 27 Gα, 5 Gβ, and 13 Gγ subunits, while plants exhibit a more streamlined G-protein system. In *Arabidopsis*, for instance, there is a single Gα subunit gene (*AtGPA1*), three XLG genes (*AtXLG1*, *AtXLG2*, *AtXLG3*), one Gβ gene (*AtAGB1*), and three Gγ genes (*AtAGG1*, *AtAGG2*, *AtAGG3*) [8,9,10]. Similarly, rice harbors one Gα subunit gene (*OsRGA1*), four XLG genes (*OsXLG1*, *OsXLG2*, *OsXLG3*, *OsXLG4*), one Gβ gene (*OsRGB1*), and five Gγ subunits (*OsRGG1-2*, *OsDEP1*, *OsGS3*, *OsGGC2*) [11,12,13,14].

G-proteins play multiple roles in plant growth and development, defense against pathogens and disease, and hormone signaling transduction, which have been well-studied in previous studies [15,16]. For instance, the Gα subunit can spontaneously release GDP, which can stabilize a GTP-bound state. However, the Gβ subunit is tightly bound to the Gγ subunit, which is known to as a functional βγ complex that modulates the activity of multiple effectors. On the other hand, recent research has reported that G-proteins are involved in response to biotic and abiotic stresses [17,18,19]. In *Arabidopsis*, a Gα protein AtGPA1 modulates drought tolerance by regulating stomatal closure, while in rice, the Gα subunit displayed a negative effect on drought tolerance [20,21]. As for the Gβ subunit, *Arabidopsis AtAGB1* enhanced salt tolerance by mitigating Na^+^ uptake in roots, while rice *OsRGG1* mediated salt responses via reactive oxygen species (ROS) scavenging [22]. It is also reported that G-protein might participate in the regulation of calcium signaling in plants [23]. For instance, Kan et al. (2021) revealed that rice G-protein negatively regulates heat tolerance by regulating wax synthesis through calcium signaling [24]. More importantly, a recent study highlighted the importance of *AT1*, a Gγ subunit, in modulating sorghum tolerance in response to salinity and alkaline stresses [25]. In addition, more attempts are still needed to elucidate the members and their function in abiotic stresses.

Barley (*Hordeum vulgare* L.) ranks as the fourth-largest cereal crop globally and is one of the earliest domesticated crops, well-known for its adaptability to harsh environments [26]. Due to its widespread use as animal feed and beer malting, barley is increasingly recognized for its nutritional benefits [27]. Wild barley, with its extensive ecological range, serves as a reservoir of genetic diversity, offering potential for improving stress resilience in cultivated varieties [28,29]. Given its status as a diploid cereal model, barley is an ideal species for genetic studies, especially with the recent release of a high-quality reference genome (MorexV3), providing a foundation for functional genomics research [30]. Despite this, no comprehensive studies on the G-protein gene family in barley have been reported.

In this study, we identified and characterized eight heterotrimeric G-protein genes from the barley reference genome (MorexV3). After performing a combination of bioinformatics approaches, we analyzed the physicochemical properties, phylogeny, chromosomal localization, gene structure, conserved motifs, cis-regulatory elements, collinearity, and protein-protein interaction networks of G-protein genes. Furthermore, we investigated their expression patterns in root and shoot tissues under salt, drought, and waterlogging stress conditions. Our findings provide new insights into the potential roles of G-protein genes in response to abiotic stress in barley, laying the groundwork for future functional studies and molecular breeding.

## 2. Results

### 2.1. Identification and Characterization of Heterotrimeric G-Protein Family Genes in Barley

BLASTp alignment and HMM searches based on *Arabidopsis* and rice G-protein family protein sequences were performed to identify eight heterotrimeric G-protein genes in the barley genome (Table 1). These G-protein genes encode proteins with considerable variation in sequence length, ranging from 100 to 910 amino acids. Among them, *HvGγ2* was identified as the shortest protein, while *HvXLG3* was the longest. Correspondingly, the molecular weights ranged from 1.11 to 10.21 kDa, with predicted isoelectric points between 4.87 and 8.33. Those proteins also displayed a wide range of instability index values (26.09–81.70), suggesting differential stability under physiological conditions. Meanwhile, their grand average hydropathy (GRAVY) indices ranged from −0.103 to 1.188, indicating variability in hydrophobicity. However, none of the G-proteins exhibited signal peptides or transmembrane domains, consistent with their predicted non-secretory nature. Subcellular localization analysis also revealed that seven of the proteins are likely nuclear-localized, while only *HvGα1* is predicted to reside in the cytoplasm. This suggests diverse functional roles for these G-proteins in intracellular signaling and cellular compartmentalization in barley (Table 1).

### 2.2. Phylogeny and Gene Ontology (GO) Analysis of G-Protein Genes

**Figure 1:**
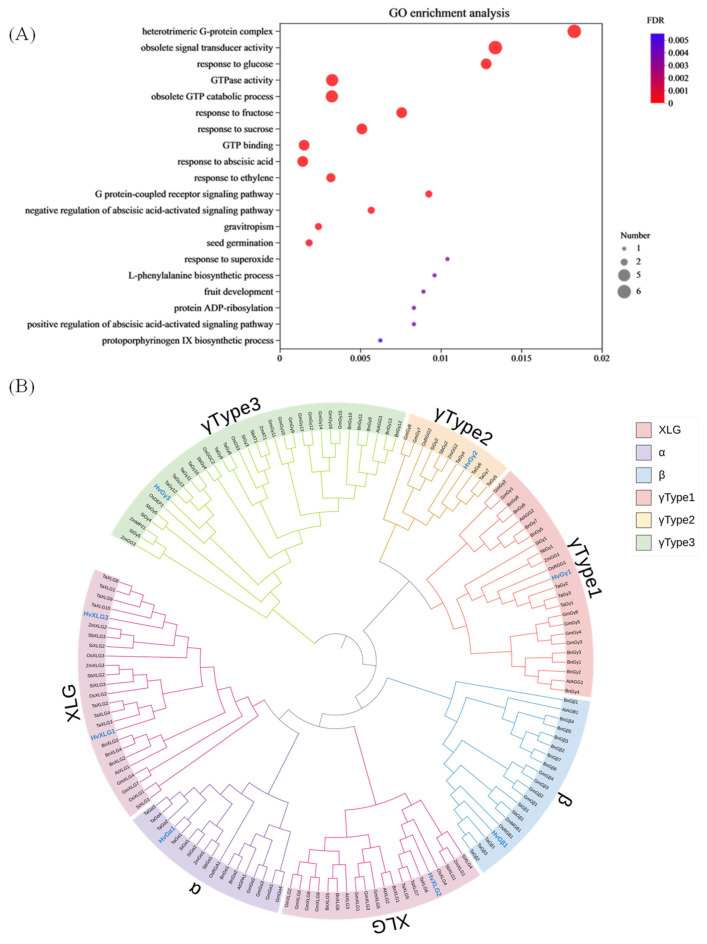
Phylogeny and gene ontology (GO) analysis of G-protein genes. (**A**) Number of G-protein family genes in diploid and polyploids of different species; (**A**) gene ontology (GO) annotation and GO enrichment analysis of G-protein genes; (**B**) phylogenetic tree of G-protein family in different crop species, including barley, *Arabidopsis*, rice, maize, soybean, sorghum, wheat, rapeseed, and millet.

To investigate the evolutionary relationships among the identified G-protein genes, a comprehensive phylogenetic analysis was conducted across several plant species. A total of 8, 8, 11, 10, 27, 10, 31, 28, and 12 heterotrimeric G-protein genes were identified in barley, *Arabidopsis*, rice, maize, soybean, sorghum, wheat, rapeseed, and millet, respectively (Figure S1). Meanwhile, gene ontology (GO) classification provided insights into the barley G-protein gene set. Most of these G-protein genes were linked to a diverse range of biological processes, including responses to superoxide, ethylene, abscisic acid, and regulation of the mitotic cell cycle. Interestingly, six genes were annotated with the term “obsolete GTP catabolic process”, indicating the potential role of these genes in signal transduction pathways. In terms of molecular function, categories such as “G-protein beta/gamma-subunit complex binding”, “GTP binding”, and “GTPase activity” were prominent, with six genes associated with the term “obsolete signal transducer activity”. The cellular component annotations highlighted that six genes localized to the “heterotrimeric G-protein complex”, and others related to the nucleus, underscoring their involvement in intracellular signaling pathways (Figure 1A). Subsequently, these G-protein genes were partitioned into four subfamilies in nine plant species. The phylogenetic analysis revealed that barley G-protein family members form well-defined clusters with their orthologs in monocots, consistent with the current understanding of plant evolutionary history (Figure 1B, Table S1). The phylogenetic tree, constructed using G-protein homologs from *Arabidopsis*, rice, and barley, further confirmed that barley G-proteins are more closely related to monocot species. This finding aligns with the established monocot-dicot evolutionary framework. Specifically, the Gγ subfamily exhibited diversification into three distinct types, including type1, type2, and type3, the latter two being unique to plants (Figure S2).

### 2.3. Gene Structure, Conserved Motifs, Domains, and Chromosomal Localization of G-Protein Family Members in Barley

**Figure 2:**
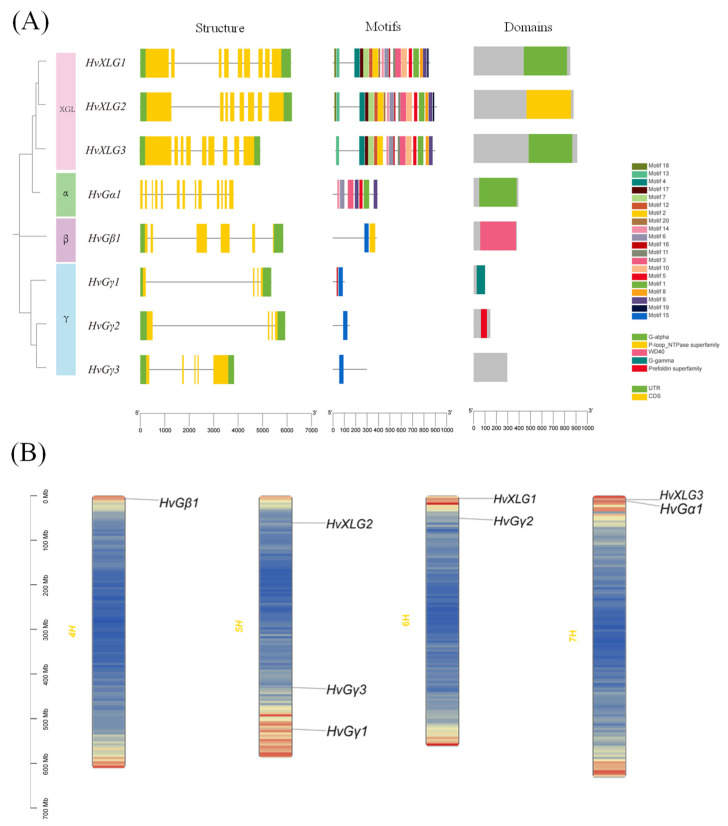
Gene structure, conserved motifs, domains, and chromosomal localization of G-protein family members in barley. (**A**) Analysis of gene structure (left), conserved motifs (middle), and domains (right) of G-protein family in barley; (**B**) chromosome location maps of G-protein genes in barley. The left side scale bar is for the physical length of chromo-some (Mb).

Barley G-protein genes have completely different exon-intron structure, while some similarity exists within subfamilies (Figure 2A). They can be categorized into four subfamilies: Gα, Gβ, Gγ, and XLG. Notably, the Gα (*HvGα1*) and Gβ (*HvGβ1*) subfamilies are represented by a single gene, each comprised 13 and 6 exons, respectively. In the Gγ subfamily, *HvGγ1* and *HvGγ2* contain four exons, while *HvGγ3* harbors five exons. In contrast, the XLG subfamily members (*HvXLG1*, *HvXLG2*, and *HvXLG3*) contain eight to nine exons. A unique feature of the XLG protein is the relay on the C-terminal region, which shares significant sequence homology with the Gα subfamily, showing a potential evolutionary link. Additionally, both Gβ and Gγ subfamilies exhibited conserved coiled-coil motifs, facilitating the formation of Gβγ dimers. These findings support the phylogenetic analysis based on the homology of Gα(G-alpha), Gβ(WD40), and Gγ(GGL) (Figure 2A).

Motif analysis using MEME method identified 20 distinct motifs (motifs 1–20) in barley G-protein family. Notably, the XLG subfamily shares a highly conserved motif composition with *HvGα1*, suggesting their close phylogenetic relationship. The Gβ and Gγ subfamilies, on the other hand, share motif 15, relative to the functional homology (Figure 2A).

Chromosomal localization analysis revealed that the barley G-protein genes are unevenly distributed across chromosomes 4, 5, 6, and 7. Chromosome 5 harbors the highest number, with three genes, while chromosomes 6 and 7 each contain two genes, and chromosome 4 contains a single G-protein gene (Figure 2B).

### 2.4. Collinearity, Selection Pressure Analysis, and Tertiary Structure Prediction of Heterotrimeric G-Protein Family in Barley

**Figure 3:**
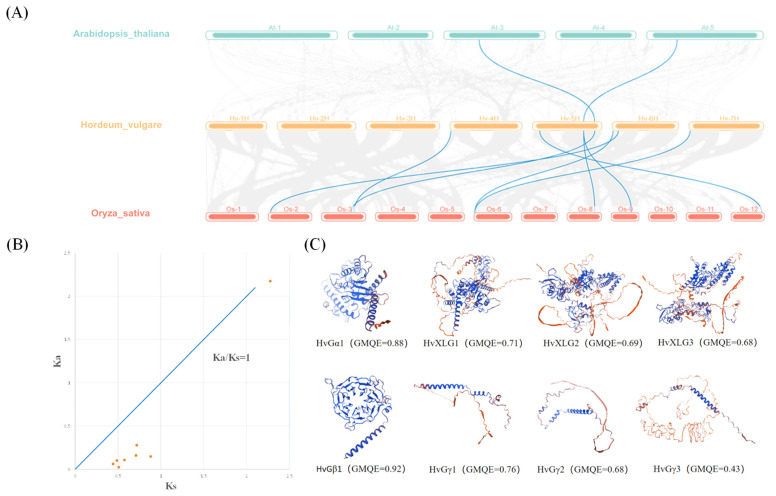
Collinearity, selection pressure analysis, and tertiary structure prediction of heterotrimeric G-protein family in barley. (**A**) Collinearity diagram of barley G-protein with *Arabidopsis thaliana* and rice.Gray lines: all synteny blocks in the *H. vulgare* genome. Blue lines: duplicated gene pairs; (**B**) Ka/Ks ratios of duplicated pairs of G-protein in barley; (**C**) the 3D structure modeling of G-protein in barley; (**C**) The 3D structure modeling of G-protein in barley. The pymol software (3.1.3) was used to create the structural image.

Collinearity analysis between barley, *Arabidopsis*, and rice uncovered greater homology between barley and rice G-protein genes compared to *Arabidopsis*, with eight orthologous gene pairs identified in rice and only two in *Arabidopsis* (Figure 3A). This suggests a closer evolutionary relationship between monocot species, consistent with the phylogenetic positioning of barley.

To further understand the evolutionary dynamics, we conducted a selection pressure analysis by calculating the non-synonymous (Ka) and synonymous (Ks) substitution rates, as well as the Ka/Ks ratios (Table 2). A Ka/Ks ratio of less than 1 indicates purifying selection, whereas ratios near 1 suggest neutral evolution, and ratios greater than 1 signify positive selection. The majority of the identified gene pairs exhibited Ka/Ks ratios below 0.9, indicating strong purifying selection pressure. However, the *OsXLG4/HvXLG2* gene pair showed a Ka/Ks ratio of 0.95, suggesting moderate sequence divergence while still under purifying selection (Figure 3B).

Three-dimensional structural predictions revealed that eight barley G-protein genes differ in overall structure and significant similarities (Figure 3C). The C-terminal of XLG and Gα subfamily genes are conserved, featuring CD domains with critical functional regions such as switch 1, switch 2, and switch 3, which are involved in GTP binding and hydrolysis. The Gα subfamily also contains a helical domain and a P-loop motif at the N-terminus, whereas the XLG subfamily is distinguished by a cysteine-rich sequence. The Gβ subfamily proteins are characterized by seven WD40 repeats at the C-terminus, contributing to their β-propeller structure, while the Gγ subfamily proteins exhibit N-terminal coiled-coil motifs that facilitate dimerization with Gβ subunits. Interestingly, Type A Gγ proteins possess palmitoylation sites and a CaaX prenylation motif (CXXL), while Type B Gγ proteins lack additional structural elements at the C-terminus. In contrast, Type C Gγ proteins contain a transmembrane helix and a cysteine-rich region, contributing to their extended length.

### 2.5. Stress-Related Cis-Elements in the Promoter of Heterotrimeric G-Protein Regulatory Genes in Barley

**Figure 4:**
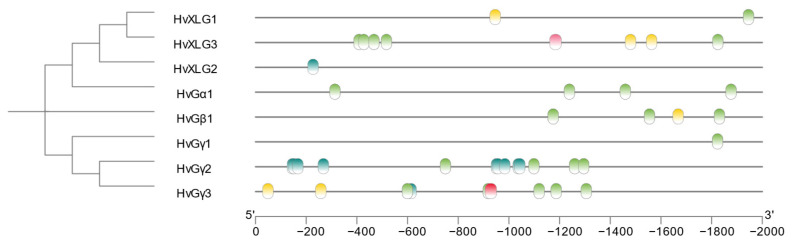
The homeostatic cis-elements of G-protein regulatory genes in barley.

Cis-regulatory elements play a pivotal role in modulating gene expression under various environmental and physiological conditions. To elucidate the regulatory mechanisms governing the expression of G-protein genes in barley, we conducted an in silico analysis of their promoter regions (2 kb upstream of the coding sequences) to identify stress-related cis-elements. The analysis uncovered a diverse array of cis-elements associated with responses to abiotic stress and hormone signaling pathways (Figure 4). Specifically, five key cis-elements, including those responsive to methyl jasmonate (MeJA), light, low-temperature, abscisic acid (ABA), and the ABA signaling pathway, were prominently featured in the promoter regions, highlighting their potential role in stress adaptation.

Among the G-protein genes, the promoter region of *HvGγ2* harbored the highest number of cis-elements, containing 13 distinct motifs related to stress responses. In contrast, the promoter regions of *HvXLG2* and *HvGγ1* exhibited the fewest cis-elements, with only one stress-related motif each. Additionally, the promoter of *HvGγ3* contained four distinct types of cis-elements, underscoring its involvement in multiple stress response pathways. These findings indicate that members of the G-protein gene family may participate in a wide range of biological processes, particularly in response to abiotic stress and hormonal regulation, contributing to their functional diversity under stress conditions.

### 2.6. Functional and Regulatory Network of Heterotrimeric G-Protein Genes in Barley

**Figure 5:**
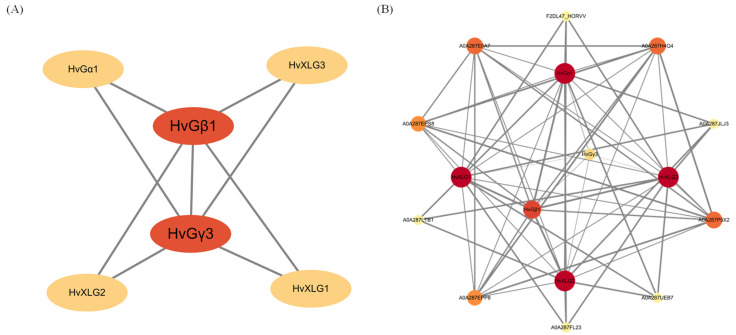
Functional and regulatory network of heterotrimeric G-protein genes in barley. (**A**) Protein–protein interaction (PPI) network of G-protein in barley; (**B**) predictive protein interaction analysis of G-protein in barley. Gray connecting lines represent the predicted protein interactions, with the color gradually increasing from dark (red) to light (yellow), indicating a gradual increase in the number of interacting genes.

To further elucidate the functional roles of heterotrimeric G-proteins in barley, a protein-protein interaction (PPI) network was constructed using the STRING database (Figure 5). The analysis revealed that G-protein family members may interact not only with one another but also with a broad spectrum of other proteins involved in various biological processes, including signal transduction, gene regulation, energy metabolism, and cell cycle control.

Specifically, β and γ subunits form dimers that may interact with α subunits to establish functional heterotrimers, which are critical for G-protein signaling. Beyond the formation of G-protein heterotrimers, several interactions were identified with proteins involved in the ubiquitin-proteasome pathway, including “ubiquitin carboxyl-terminal hydrolase” and “UCH_1 domain-containing proteins”. Moreover, connections with key regulators of lipid metabolism, such as “phosphoinositide phospholipase C”, suggest a role for G-proteins in the modulation of phosphoinositide signaling (Table S3). These interactions underscore the multifunctional nature of G-protein complexes in barley, potentially regulating diverse physiological processes that span signal transduction, proteostasis, and metabolic pathways.

### 2.7. Expression Patterns of Heterotrimeric G-Protein Regulatory Genes in Different Tissues and Under Abiotic Stresses

**Figure 6:**
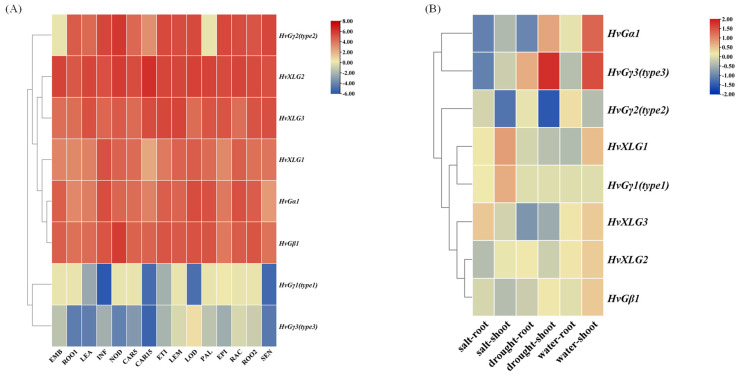
Expression patterns of heterotrimeric G-protein regulatory genes in different tissues and under abiotic stresses. (**A**) Expression heatmap of G-protein during barely development in different seedling tissues in the database. Note: EMB: Germinating embryos after 4 days; ROO1: roots from seedlings; LEA: shoots from seedlings; INF: developing inflorescences (1 cm); NOD: developing tillers, 3rd internode; CAR5: developing grain (5 days after pollination); CAR15: developing grain (15 days after pollination); ETI: etiolated seedling, dark cond (dark treatment for 10 days); LEM: inflorescences, lemma (42 days after pollination); LOD: inflorescences, lodicule (42 days after pollination); PAL: dissected inflorescences, palea (42 days after pollination); EPI: epidermal strips (28 days after pollination); RAC: inflorescences, rachis (35 days after pollination); ROO2: roots (28 days after pollination); SEN: senescing leaves (56 days after pollination); (**B**) expression heatmap of G-protein during barely development under salt, drought, and waterlogging conditions.

The expression patterns of heterotrimeric G-protein genes were examined across various tissues and in response to different abiotic stress conditions using transcriptome data from public databases and experimental datasets generated by our group. The analysis revealed that six G-protein genes were significantly expressed across a range of tissues, whereas two genes *HvGγ1* and *HvGγ3* expressed to a lesser extent (Figure 6A). Specifically, we observed that *HvGα1*, *HvGγ2*, and *HvGγ3* displayed distinct expression patterns compared to other G-protein genes, respectively, under salt, drought, and waterlogging stresses (Figure 6B), indicating their involvement in stress response mechanisms. Specifically, *HvGα1* and *HvGγ3* exhibited marked upregulation (Log_2_FC = 1.33, 1.54) under waterlogging stress conditions in shoot, *HvGγ3* exhibited marked upregulation (Log_2_FC = 1.82) under drought stress conditions in shoot, *HvGγ2* exhibited marked downregulation (Log_2_FC = 1.53, 1.20) under both drought and salt conditions in shoot, *HvGα1* and *HvGγ3* exhibited marked downregulation (Log_2_FC = 1.06, 1.05) under salt stress conditions in root. This result suggests their prominent roles in both barley development and stress adaptation. The pronounced responsiveness of *HvGγ3* to abiotic stress highlights their potential regulatory roles in barley’s stress resilience (Figure 6B).

### 2.8. Validation of Expression Patterns Using qRT-PCR

**Figure 7:**
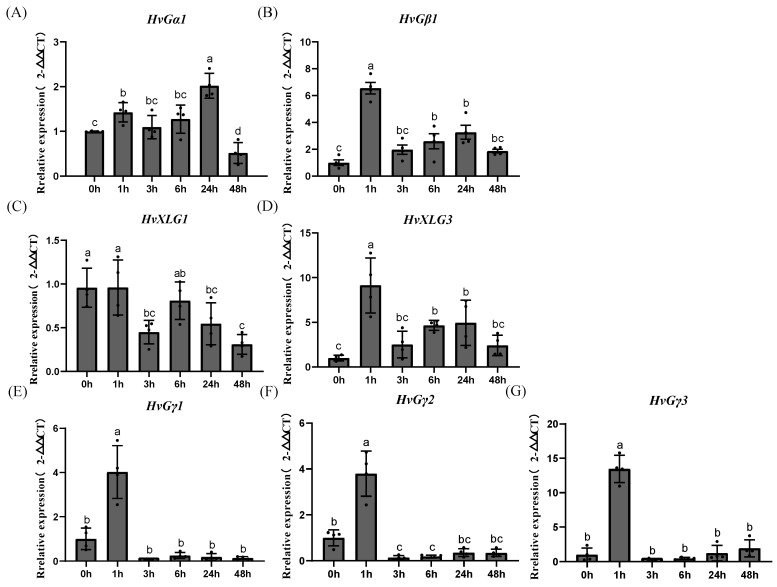
Expression patterns of selected seven G-protein genes in barley under salt stress. Error bars indicate the standard deviations of at least three biological replicates, and different letters show significant differences using the LSD test at *p* < 0.05.

To further explore the response patterns under salt stress in a short time, seven genes were selected for detailed qRT-PCR analysis (Figure 7). Under short-term salt stress, the γ subfamily genes exhibited similar expression patterns, with no significant changes observed for HvGα1, which only reached its lowest expression level at 48 h. Conversely, *HvGβ1*, *HvXLG2*, *HvGγ1*, *HvGγ2*, and *HvGγ3* peaked at 1 h post-treatment. The rapid increase in *HvGγ3* expression, with a notable peak at 1 h, suggests that *HvGγ3* may play a crucial role in early-stage responses to abiotic stress in barley. This early response, coupled with the elevated expression levels, underscores *HvGγ3*’s potential as a key player in stress resilience pathways.

## 3. Discussion

The first α subunit of G-protein, *AtGPA1*, was identified and cloned in *Arabidopsis thaliana* by Ma et al. in 1990 [16], marking the beginning of studies on G-proteins in plants. Since that pivotal discovery, the field has been expanded significantly with notable differences in G-protein signaling between animals and plants. In animals, a diverse array of G-protein subunits and G-protein-coupled receptors (GPCRs) contributes to a wide variety of G-protein signaling pathways [31]. Conversely, plants possess a more limited repertoire of G-protein subunits. The Gα subunit in plants can spontaneously release GDP, transitioning to a GTP-bound state that plays a crucial role in regulating growth and developmental processes [32].

Despite advances in biotechnology that have facilitated extensive research on animal G-proteins, plant G-proteins—particularly the unique extra-large G-proteins (XLG) and atypical Gγ subunits—remain less well characterized [33]. Recent bioinformatics analyses have identified G-protein-related genes across various species, providing insight into their evolutionary conservation and functional divergence. In this study, we employed bioinformatics methods to identify eight G-protein genes within the barley genome. This number reflects a relatively small G-protein gene family, compared to other plant species. For instance, *Arabidopsis* contains five G-protein genes [34], while rice has seven [35]. In contrast, soybean, which is a dicotyledonous plant, exhibits 18 G-protein genes due to genomic duplication events [36], and wheat also possesses 18 G-protein genes [37]. Notably, three large Gα homologous genes (XLG) have been identified in *Arabidopsis* [38] and two in rice [35], illustrating the variability in G-protein family sizes among different plant species.

Phylogenetic analysis revealed that the G-protein gene family members from nine crops can be classified into four distinct subunits. Among these, the γ subfamily can be further subdivided into three types [39], highlighting the complexity and evolutionary relationships within the G-protein family. The analysis demonstrated that the protein sequence homology among genes in subfamilies, except for the γ subfamily, was relatively high, suggesting conserved functions.

The classification of G-protein genes into four groups was supported by phylogenetic trees, motif analyses, and gene structure evaluations. Here, eight G-protein genes comprise one from the β subfamily, three from the γ subfamily, three from the XLG subfamily, and one from theαsubfamily in barley. Subcellular localization predictions indicated that all G-protein genes, with the exception of *HvGα1*, are localized in the nucleus, implicating a role in transcriptional regulation and signal transduction within this organelle. Gene Ontology (GO) analysis indicated these genes are likely involved in processes related to growth, development, and stress responses in barley. Notably, the similar relation of barley G-proteins with their wheat counterparts suggests potential functional similarities within subfamilies that needs further investigation.

The difference or similarity in exon-intron patterns among G-protein genes within the same subfamily may reflect evolutionary pressures and functional constraints. The motif analysis revealed that the α and XLG subfamilies share similar motifs, while the β and γ subfamilies exhibit distinct motif compositions. This distinctiveness might reveal the special roles of these proteins in signaling pathways. As predicted, the XLG proteins are approximately twice the length of Gα proteins and share significant homology in their C-terminal regions with Gα proteins. However, they are characterized by distinct N-terminal regions containing regularly spaced cysteine residues and nuclear localization signals (NLS) [40,41]. In the model bryophyte *Physcomitrella patens*, which lacks Gα subunits but contains XLG, Gβ, and Gγ, XLGs may evolve independently from Gα subunits [42]. The XLGs possess an N-terminal domain similar to classic Gγ (type 1) subunits alongside a C-terminal domain enriched in cysteine residues [43]. The evolutionary trajectories of XLGs and atypical Gγ subunits probably contribute to a compact yet complex G-protein regulatory system within plants.

Cis-acting element analysis of the promoter regions of G-protein genes in barley revealed an array of elements associated with stress responses and regulatory processes [44]. Key elements responsive to methyl jasmonate (MeJA), MYB binding sites, light, low temperatures, and abscisic acid (ABA) signaling pathways were identified. These findings suggest that G-protein genes may play integral roles in plant growth, development, and stress responses, particularly in coping with abiotic stressors such as salinity and drought.

Transcriptome data indicated that, except for *HvGγ1* and *HvGγ3*, most G-protein genes were significantly expressed across various tissues. Under stress conditions, *HvGγ3* exhibited a down-regulated expression in roots under salt stress but was up-regulated in shoots during drought stress. This differential regulation highlights the potential significance of *HvGγ3* as a key gene in the response to salt stress, a hypothesis that is supported by quantitative reverse transcription PCR (qRT-PCR) results.

The protein-protein interaction (PPI) network analysis suggested that G-protein genes interact with proteins implicated in various pathways, including “ubiquitin carboxyl terminal hydroxylase”, “UCH_1 domain containing protein”, and “phosphoinositide phospholipase C”. These interactions imply that G-proteins may regulate these pathways, thus contributing to their roles in stress responses and other biological processes. In addition to the aforementioned proteins, several others—such as *AtGPA1* [45], *OsRGB1* [46], *NDL1* [47], and *COLD1* [23]—have been reported to be involved in various growth and developmental processes as well as in plant stress responses across different species.

Current studies indicate that G-proteins, including the *AT1*-encoded Gγ gene, can significantly influence plant tolerance to both salt and alkaline conditions [25]. G-proteins are also related to calcium signaling, with recent findings establishing a link between G-proteins, Ca^2+^ sensing, and wax metabolism, thereby identifying key genes for heat tolerance in crops [24]. While the number of plant heterotrimeric G-protein families is smaller than that of animals, their regulatory roles in abiotic stress responses are nonetheless crucial. More importantly, G-proteins engage in multiple signaling pathways, with notable intersections between different pathways, complicating the understanding of their detailed mechanisms of action.

There remains a significant gap in understanding the intricate pathways of G-proteins. Our research provides some candidate G-protein genes for further exploration of plant stress mechanisms, while the functional validation of these genes is essential in future research. With advancements in molecular biology, bioinformatics, and crop science, we predicted the specific functions of these G-protein genes. Overall, these results provided valuable insights for future research on G-protein genes and the exploration of gene resources involved in plant stress responses.

## 4. Materials and Methods

### 4.1. Genome-Wide Identification and Physicochemical Analysis of G-Protein Gene Family Members in Barley

Whole genome data, including coding sequences (CDSs), protein sequences, and annotation files for barley, rice, and *Arabidopsis* were downloaded from the Ensemble Plants database (https://plants.ensembl.org/index.html (accessed on 20 March 2024)). The amino acid sequences of G-proteins of rice and *Arabidopsis* were used as reference sequences and searched using BLASTp (e < 1 × 10^−20^). Hidden Markov models (HMMs) of the G-protein domains—Gα (PF00503), Gβ (PF00400), and Gγ (PF00631)—were obtained from the Pfam database (http://pfam.sanger.ac.uk/ (accessed on 20 March 2024)) (*p* < 0.001). Subsequently, the online tools CDD-search (https://www.ncbi.nlm.nih.gov/cdd/ (accessed on 20 March 2024)) was employed to identify genes with the corresponding conserved domains, determining the final members of the gene family. Sequences were verified using Pfam and SMART to confirm the presence of conserved Gα (G-alpha), Gβ (WD40), and Gγ (GGL) domains, and gene members were categorized accordingly.

The physicochemical properties of G-proteins, including amino acid number, isoelectric point, signal peptide, instability coefficient, and hydrophilicity, were predicted using the ExPASy ProtParam tool website (https://web.expasy.org/protparam/ (accessed on 22 March 2024)), and the parameters were set as default values. Subcellular localization predictions were made using online tool WoLF PSORT (https://wolfpsort.hgc.jp/ (accessed on 22 March 2024)).

### 4.2. Phylogenetic Analysis and Gene Ontology (GO) Annotation Analysis of the Barley G-Protein Gene Family

The selected eight G-protein genes in barley were annotated by Gene Ontology (GO) database. GO enrichment can be divided into three categories: biological processes, cellular components, and molecular functions. The obtained results were plotted on the Majorbio platform (https://www.majorbio.com/ (accessed on 18 July 2024)).

Multiple sequence alignments were performed using Clustal X (version 2.1) across G-protein gene families from barley, *Arabidopsis*, rice, maize, soybean, sorghum, wheat, rapeseed, and millet to explore evolutionary relationships among these species. Phylogenetic trees were constructed using the NJ method with 1000 bootstrap replications via MEGA11 (version 11.0.13) software and FastTree, and visualized using online tool iTOL (https://itol.embl.de (accessed on 20 March 2024)).

### 4.3. Gene Structure and Collinearity Analysis of the Barley G-Protein Gene Family

Intron and exon characteristics of the G-protein genes in barley were analyzed using TBtools-II, based on gene annotations. G-protein motifs were identified using the MEME online tool (http://meme.sdsc.edu/meme/ (accessed on 24 March 2024)), and both gene structure and conserved motifs were visualized with TBtools-II (version 2.056).

Collinearity data were obtained using MCScanX, and both segmental and tandem duplication events were identified within the barley genome (MorexV3). Since no collinearity was observed within the gene family members in barley but was present between species, non-synonymous (Ka) and synonymous (Ks) substitution rates and Ka/Ks values were calculated using the Simple KaKs Calculator, based on protein sequences and CDSs of the G-protein gene family. Collinear diagrams were visualized using TBtools-II.

### 4.4. Gene Promoter, Chromosome Mapping, and Three-Dimensional Structure of the G-Protein Gene Family in Barley

Chromosome localization of the G-protein gene family was visualized using TBtools-II software, based on gene annotations file of the barley genome. Protein homology modeling and three-dimensional structure predictions were carried out using online tool Swiss-Model (https://swissmodel.expasy.org (accessed on 28 March 2024)). Promoter sequences (2 kb upstream of the CDS region) for G-protein genes were extracted using TBtools-II (version 2.056). Cis-elements were predicted using the PlantCARE database (http://bioinformatics.psb.ugent.be/webtools/plantcare/html/ (accessed on 28 March 2024)), which analyzed the stress and hormone response elements.

### 4.5. Protein Interaction Network Analysis of the G-Protein Family Genes in Barley

The protein-protein interaction (PPI) network of G-proteins in barley was constructed using the search tool for the retrieval of interacting Genes/Proteins STRING database (https://string-db.org/ (accessed on 17 June 2024)). According to the protein-protein interaction score (≥ 0.400), the thicker line between the targets in the interaction network, the stronger the interaction will be. The results were imported into Cytoscape (v3.10.1) software to visualize the protein-protein interaction network.

### 4.6. Analysis of Expression Patterns of G-Protein Regulatory Genes

Finally, we identified expression patterns of G-protein genes in barley in ten different tissues, including EMB, ROOL, LEA, INF, NOD, CAR5, CARL5, ETI, LEM, LOD, PAL, EPI, RAC, ROO2, SEN. Transcriptome data from 15 different tissues of barley (cultivar Morex) were obtained from the APEX online resource (https://apex.ipk-gatersleben.de/apex/f?p=284) (accessed on 7 August 2024). Using the transcriptome data of roots and leaves under salt stress (100 mmol L^−1^ NaCl for 7d) [48], drought stress (7 days of drought treatment to reduce soil water content to 16−20%) [49], and waterlogging stress (water level 2 cm above the soil for 15d) [50] of barley seedlings was measured. The expression values were calculated by log2 (FPKM) and were displayed as a heat map by TBtools-II (version 2.056) software. Fold change greater than 1 represents upregulation, and fold change less than −1 represents downregulation.

### 4.7. Plant Material and Salt Stress Treatment

Barley (Golden Promise) seeds were germinated and subjected to 200 mmol L^−1^ NaCl stress. The plants were grown in an artificial climate chamber under a 16-h light period at 24 °C and an 8-h dark period at 20 °C. Root tips were collected at various time points (0, 1, 3, 6, 24, and 48 h) following salt stress. All samples were immediately flash-frozen in liquid nitrogen and stored at −80 °C for RNA extraction.

### 4.8. qRT-PCR Analysis

Seven G-protein genes, whose expression levels increased under salt treatment, were selected to verify RNA-seq data via qRT-PCR experiments. Total RNA was extracted using the Easy-Do Plant Total RNA Rapid Extraction Kit, and concentrations were quantified using an Agilent 2100 Bioanalyzer (Agilent Technologies, Santa Clara, CA, USA). cDNA synthesis was performed using HiScript III RT SuperMix for qPCR (+gDNA wiper) (Vazyme, Nanjing, China). qRT-PCR reactions were prepared according to the iQ™ SYBR Green Supermix (Bio-Rad, Hercules, CA, USA) instructions and conducted using a Roche LightCycler 480 II instrument. Amplification and melting curves were used to assess PCR dynamics and specificity, respectively. Primer sequences are listed in Supplementary Table S4, with the β-Actin gene used as an internal reference. Each treatment and reaction was performed in triplicate. Relative expression levels were calculated using the 2^−ΔΔCT^ method.

## 5. Conclusions

In this study, we conducted a comprehensive identification and analysis of eight G-protein gene family members within the barley genome. We further investigated their physicochemical properties, evolutionary relationships, gene structures, protein motif compositions, three-dimensional (3D) structural features, gene duplication events, chromosomal distributions, and cis-acting regulatory elements. Moreover, we examined their expression profiles under abiotic stress conditions, including salt, drought, and waterlogging stresses at various time points. Notably, expression analysis revealed that *HvGγ3* gene exhibited a significant and dynamic response to salt stress, highlighting its potential role in regulating stress tolerance in barley, which is particularly crucial for crop productivity in increasing salinity soils. These findings provide a foundation for future investigations in the intricate regulatory networks of G-proteins in stress tolerance, and also provide strategies for molecular breeding with high stress resistance.

## Figures and Tables

**Table 1:** Basic information of G-protein regulatory gene members in barley.

Gene Name	Gene ID	Amino Acid Number(aa)	Isoelectric Point	Molecular Weight (KDa)	Prediction of Subcellular Localization	Unstability Index	Grand Average of Hydropathicity
*HvGα1*	HORVU.MOREX.r3.7HG0641160.1	393	5.84	45.35	Cytoplasm	36.65	−0.464
*HvXLG1*	HORVU.MOREX.r3.6HG0540400.1	849	5.29	95.92	Nucleus	55.79	−0.596
*HvXLG2*	HORVU.MOREX.r3.5HG0437540.1	875	5.23	95.90	Nucleus	58.66	−0.355
*HvXLG3*	HORVU.MOREX.r3.7HG0639080.1	910	5.36	102.10	Nucleus	56.14	−0.635
*HvGβ1*	HORVU.MOREX.r3.4HG0333760.1	380	7.13	41.70	Nucleus	26.09	−0.262
*HvGγ1 (type1)*	HORVU.MOREX.r3.5HG0509020.1	100	5.43	11.15	Nucleus	65.39	−0.541
*HvGγ2 (type2)*	HORVU.MOREX.r3.6HG0556710.1	146	4.87	16.29	Nucleus	81.70	−1.188
*HvGγ3 (type3)*	HORVU.MOREX.r3.5HG0480200.1	295	8.33	31.57	Nucleus	76.12	−0.103

**Table 2:** Gene replication information regulated by barley heterotrimer G-protein.

Homologous Gene Pair		Ka	Ks	Ka/Ks	EffectiveLen	AverageS-sites	AverageN-sites	cN	cS	pN	pS
Gene1	Gene2										
*AtAGG1*	*HvGγ1*	0.36	NaN	NaN	300.00	68.42	231.58	65.50	60.50	0.28	0.88
*AtAGG3*	*HvGγ3*	0.50	NaN	NaN	642.00	145.92	496.08	179.92	113.08	0.36	0.77
*OsRGG2*	*HvGγ2*	0.10	0.49	0.21	405.00	93.42	311.58	29.50	33.50	0.09	0.36
*OsRGB1*	*HvGβ1*	0.02	0.51	0.05	1140.00	273.42	866.58	20.00	101.00	0.02	0.37
*OsRGG1*	*HvGγ1*	0.06	0.44	0.14	279.00	63.17	215.83	12.83	21.17	0.06	0.34
*OsXLG3*	*HvXLG1*	0.15	0.88	0.17	2532.00	573.83	1958.17	262.50	297.50	0.13	0.52
*OsXLG3*	*HvXLG3*	0.11	0.58	0.19	2565.00	587.42	1977.58	196.83	236.17	0.10	0.40
*OsGGC2*	*HvGγ3*	0.28	0.72	0.39	831.00	190.17	640.83	149.00	88.00	0.23	0.46
*OsDEP1*	*HvGγ3*	0.16	0.71	0.23	873.00	193.67	679.33	98.08	88.92	0.14	0.46
*OsXLG4*	*HvXLG2*	2.18	2.28	0.96	2052.00	506.00	1546.00	1095.75	361.25	0.71	0.71

Note: For the selection analysis between Arabidopsis and barley gene pairs, high sequence divergence value (pS ≥ 0.75).

## Data Availability

The data presented in this study are available on request from the corresponding author. Qiufang Shen (shenqf@zju.edu.cn).

## Supplementary Materials

The following supporting information can be downloaded at: https://www.mdpi.com/article/10.3390/plants13243521/s1, Figure S1: Number of G-protein family genes in diploid and polyploids of different species; Figure S2: Phylogenetic tree of G-protein family in barley, *Arabidopsis*, and rice; Table S1: The information of G-protein regulatory genes in barley, *Arabidopsis*, rice, maize, soybean, sorghum, wheat, rapeseed, and millet; Table S2: Characteristics of G-protein regulatory gene family in barley; Table S3: Functional proteins interact with G-proteins; Table S4: The sequences of G-protein gene primers.
