# Supplementary material for: Genome-Wide Identification and Expression Analysis of the G-Protein Gene Family in Barley Under Abiotic Stresses
Source: Plants (Basel). 2024 Dec 17;13(24):3521. doi: 10.3390/plants13243521 (PMC11677487; doi:10.3390/plants13243521)

Table S1. The information of G-protein regulatory genes in barley, *Arabidopsis*, rice, maize, soybean, sorghum, wheat, rapeseed, and millet.

species	Subfamily	Gene Name	Gene ID	Exon Number	Protein Length
Arabidopsis thaliana	*α*	*AtGPA1*	AT2G26300	14	383
	XLG	*AtXLG1*	AT1G31930	8	848
		AtXLG2	AT4G34390	8	861
		AtXLG3	AT2G23460	7	888
	β	*AtAGB1*	AT4G34460	6	377
	γ-type1	*AtAGG1*	AT3G63420	4	98
		AtAGG2	AT3G22942	4	100
	γ-type3	*AtAGG3*	AT5G20635	4	200
Oryza sativa	*α*	*OsRGA1*	Os05g0333200	13	380
	XLG	*OsXLG1*	Os10g0117800	8	828
		OsXLG2	Os11g0206700	9	856
		OsXLG3	Os06g0111400	9	867
		OsXLG4	Os12g0593000	8	896
	β	*OsRGB1*	Os03g0669200	6	380
	γ-type1	*OsRGG1*	Os03g0635100	4	93
	γ-type2	*OsRGG2*	Os02g0137800	4	150
	γ-type3	*OsGS3*	Os03g0407400	5	232
		OsGGC2	Os08g0456600	5	335
		OsDEP1	Os09g0441900	5	426
Hordeum vulgare	*α*	*HvGα1*	HORVU.MOREX.r3.7HG0641160.1	13	393
	XLG	*HvXLG1*	HORVU.MOREX.r3.6HG0540400.1	9	849

		HvXLG2	HORVU.MOREX.r3.5HG0437540.1	8	875
		HvXLG3	HORVU.MOREX.r3.7HG0639080.1	9	910
	β	*HvGβ1*	HORVU.MOREX.r3.4HG0333760.1	6	380
	γ-type1	*HvGγ1*	HORVU.MOREX.r3.5HG0509020.1	4	100
	γ-type2	*HvGγ2*	HORVU.MOREX.r3.6HG0556710.1	4	146
	γ-type3	*HvGγ3*	HORVU.MOREX.r3.5HG0480200.1	5	295
Zea mays	*α*	*ZmGα1*	GRMZM2G064732_T01		
	XLG	*ZmXLG1*	GRMZM2G127739_T01		
		ZmXLG2	GRMZM2G429113_T01		
		ZmXLG3	GRMZM2G016858_T01		
	β	*ZmMGB1*	GRMZM2G045314_T01		
	γ-type1	*ZmGG1*	GRMZM6G935329_T01		
	γ-type2	*ZmGG2*	GRMZM2G015578_T01		
	γ-type3	*ZmAT1*	GRMZM2G139878_T01		
		ZmGG3	GRMZM2G001660_T01		
		ZmMP21	GRMZM2G172320_T01		
Sorghum bicolor	*α*	*SbGα1*	SORBI_3001G484200		
	XLG	*SbGαXLG1*	SORBI_3008G150000		
		SbGαXLG2	SORBI_3004G002100		
		SbGαXLG3	SORBI_3010G009400		
	β	*SbGβ1*	SORBI_3001G142100		
	γ-type1	*SbGγ1*	SORBI_3001G161000		
	γ-type2	*SbGγ2*	SORBI_3004G035200		
	γ-type3	*SbAT1*	SORBI_3001G341700		
		SbGγ4	SORBI_3007G149200		

		SbGγ5	SORBI_3002G216600
Setaria italica	*α*	*SiGα1*	Seita.3G382500.1.p
		SiGα2	Seita.9G519700.1.p
	XLG	*SiαXLG1*	Seita.9G295300.1.p
		SiαXLG2	Seita.4G009500.1.p
		SiαXLG3	Seita.1G002600.1.p
		SiαXLG4	Seita.3G373200.1.p
	β	*SiGβ1*	Seita.9G145400.1.p
	γ-type1	*SiGγ1*	Seita.9G162300.1.p
		SiGγ2	Seita.1G093100.1.p
	γ-type3	*SiGγ3*	Seita.9G369300.1.p
		SiGγ4	Seita.2G219800.1.p
		SiGγ5	Seita.6G171500.1.p
Triticum aestivum	*α*	*TaGα1*	TraesCSU02G146500.1
		TaGα2	TraesCS1B02G479100.2
		TaGα3	TraesCS7D02G000100.3
		TaGα4	TraesCS7A02G000200.1
	XLG	*TaGαXLG1*	TraesCS7A02G011900.1
		TaGαXLG2	TraesCS6B02G012700.1
		TaGαXLG3	TraesCS6A02G007400.1
		TaGαXLG4	TraesCS6D02G011700.1

		TaGαXLG5	TraesCS5B02G068300.2
		TaGαXLG6	TraesCS5D02G075200.1
		TaGαXLG7	TraesCS5A02G064400.1
		TaGαXLG8	TraesCS7D02G030100.1
		TaGαXLG9	TraesCS7A02G033500.1
		TaGαXLG10	TraesCS4A02G455800.1
		TaGβ1	TraesCS4A02G294000.1
	β	*TaGβ2*	TraesCS4D02G017800.1
		TaGβ3	TraesCS4B02G019900.1
		TaGγ1	TraesCS5A02G377700.1
	γ-type1	*TaGγ2*	TraesCS5B02G381300.1
		TaGγ3	TraesCS5D02G387700.1
		TaGγ4	TraesCS6A02G107800.1
	γ-type2	*TaGγ5*	TraesCS6D02G095700.1
		TaGγ6	TraesCS6B02G136400.1
		TaGγ7	TraesCS6D02G095700.1
		TaGγ8	TraesCS7A02G017700.1
		TaGγ9	TraesCS7D02G015000.1
	γ-type3	*TaGγ10*	TraesCS5B02G208700.1
		TaGγ11	TraesCS4A02G474000.1
		TaGγ12	TraesCS5D02G216900.1
		TaGγ13	TraesCS5A02G215100.1
Brassica napus	*α*	*BnGα1*	BnaA09g40600D
		BnGα2	BnaC08g33050D
	XLG	*BnXLG1*	BnaA08g11230D

	BnXLG2	BnaA05g16520D
	BnXLG3	BnaC05g24110D
	BnXLG4	BnaA09g24710D
	BnXLG5	BnaA04g13660D
	BnXLG6	BnaC04g35930D
	BnGβ1	BnaA06g12310D
	BnGβ2	BnaA08g11160D
	BnGβ3	BnaC03g66080D
β	*BnGβ4*	BnaC01g03920D
	BnGβ5	BnaA01g02660D
	BnGβ6	BnaC07g45050D
	BnGβ7	BnaA03g52860D
	BnGγ1	BnaC08g32820D
	BnGγ2	BnaC04g20670D
	BnGγ3	BnaA09g40380D
γ-type1	*BnGγ4*	BnaAnng12540D
	BnGγ5	BnaA01g24300D
	BnGγ6	BnaC03g43060D
	BnGγ7	BnaCnng55440D
	BnGγ8	BnaA03g36890D
γ-type2	*BnGγ9*	BnaA03g56070D
	BnGγ10	BnaA02g05000D
γ-type3	*BnGγ11*	BnaC03g10200D
	BnGγ12	BnaA10g14860D
	BnGγ13	BnaC09g37230D

Glycine max	*α*	*GmGα*1	GLYMA_04G056600
		GmGα2	GLYMA_17G226700
		GmGα3	GLYMA_14G098000
		*GmGα*4	GLYMA_06G057000
	XLG	*GmXLG1*	GLYMA_11G123200
		GmXLG2	GLYMA_02G062300
		GmXLG3	GLYMA_12G047900
		GmXLG4	GLYMA_07G106100
		GmXLG5	GLYMA_07G163200
		GmXLG6	GLYMA_16G144600
		GmXLG7	GLYMA_09G172000
		GmXLG8	GLYMA_01G182000
		GmXLG9	GLYMA_11G060300
	β	*GmGβ1*	GLYMA_11G118500
		GmGβ2	GLYMA_12G043900
		GmGβ3	GLYMA_06G013000
		GmGβ4	GLYMA_04G013100
	γ-type1	*GmGγ*1	GLYMA_20G211400
		*GmGγ*2	GLYMA_10G178700
		GmGγ3	GLYMA_10G03610
		GmGγ4	GLYMA_10G030900

	GmGγ5	GLYMA_02G16190
	GmGγ6	GLYMA_02G143500
	GmGγ7	GLYMA_14G132400
γ-type2	*GmGγ8*	GLYMA_17G200900
	GmGγ9	GLYMA_17G182000
	GmGγ10	GLYMA_01G143300
	GmGγ11	GLYMA_07G040200
	GmGγ12	GLYMA_15G178100
γ-type3	*GmGγ13*	GLYMA_09G070100
	GmGγ14	GLYMA_17G048600
	GmGγ15	GLYMA_13G110900
	GmGγ16	GLYMA_13G110900

Table S2. Characteristics of G-protein regulatory gene family in barley.

Subfamily	Gene name	Gene ID	Chr.	Protein					Location
				Amino acids	MW(kDa)	pI	unstability index	Grand average of hydropathicity	Integral Prediction of protein location
α	*HvGα1*	HORVU.MOREX.r3.7HG0641160.1	7	393	45.35	5.84	36.65	-0.464	Cyto
XLG	*HvXLG1*	HORVU.MOREX.r3.6HG0540400.1	6	849	95.92	5.29	55.79	-0.596	Nucl
XLG	*HvXLG2*	HORVU.MOREX.r3.5HG0437540.1	5	875	95.90	5.23	58.66	-0.355	Nucl
XLG	*HvXLG3*	HORVU.MOREX.r3.7HG0639080.1	7	910	102.10	5.36	56.14	-0.635	Nucl
β	*HvGβ1*	HORVU.MOREX.r3.4HG0333760.1	4	380	41.70	7.13	26.09	-0.262	Nucl
γ-type1	*HvGγ1*	HORVU.MOREX.r3.5HG0509020.1	5	100	11.15	5.43	65.39	-0.541	Nucl
γ-type1	*HvGγ2*	HORVU.MOREX.r3.6HG0556710.1	6	146	16.29	4.87	81.7	-1.188	Nucl
γ-type3	*HvGγ3*	HORVU.MOREX.r3.5HG0480200.1	5	295	31.57	8.33	76.12	-0.103	Nucl

Table S3. Predicted proteins interact with G proteins.

Proteins	Predicted Functional Partners
A0A287FL23	Ubiquitin carboxyl-terminal hydrolase.
A0A287LTB1	UCH_1 domain-containing protein.
F2DL47_HORVV	Ubiquitin carboxyl-terminal hydrolase.
A0A287UEB7	Ubiquitin carboxyl-terminal hydrolase.
A0A287JLJ3	Ubiquitin carboxyl-terminal hydrolase.
A0A287E0A7	Phosphoinositide phospholipase C.
A0A287EPP8	Phosphoinositide phospholipase C.
A0A287EPS8	Phosphoinositide phospholipase C.
A0A287H4Q4	Phosphoinositide phospholipase C.
A0A287P5X2	Phosphoinositide phospholipase C.

Table S4. Primer sequences of G-protein for qRT-PCR.

Gene	forward primer (5'-3')	reverse primer (5'-3')
HvGα1	GTGTGGAAGTGTGCTGCAAG	GGCTTGCTGCTCTGGAAGTA
HvXLG1	CGACTTGCTTAGTTGCTCGC	CCCATAAGCCCGACTCCTTG
HvXLG3	CAATCAGGCACCCGTCTTTCC	GGTCAAAATCGCTGAACCACT
HvGβ1	ACCCGCTTGATTACAGGCTC	GGCAGCTTATACGGCCTTCA
HvGγ1	GATCCAGGCCGAGCTCAAG	CAGATCCTGCGGACCTTCAA
HvGγ2	ACTGAAGGAAAGCCTGACCC	GGAACCACCTGTCCCATGAA
HvGγ3	AGTCTCACGTTCTGCTTGCT	GCCATGAAGCACATACGCAC

Figure S1 Number of G-protein family genes in diploid and polyploids of different species.

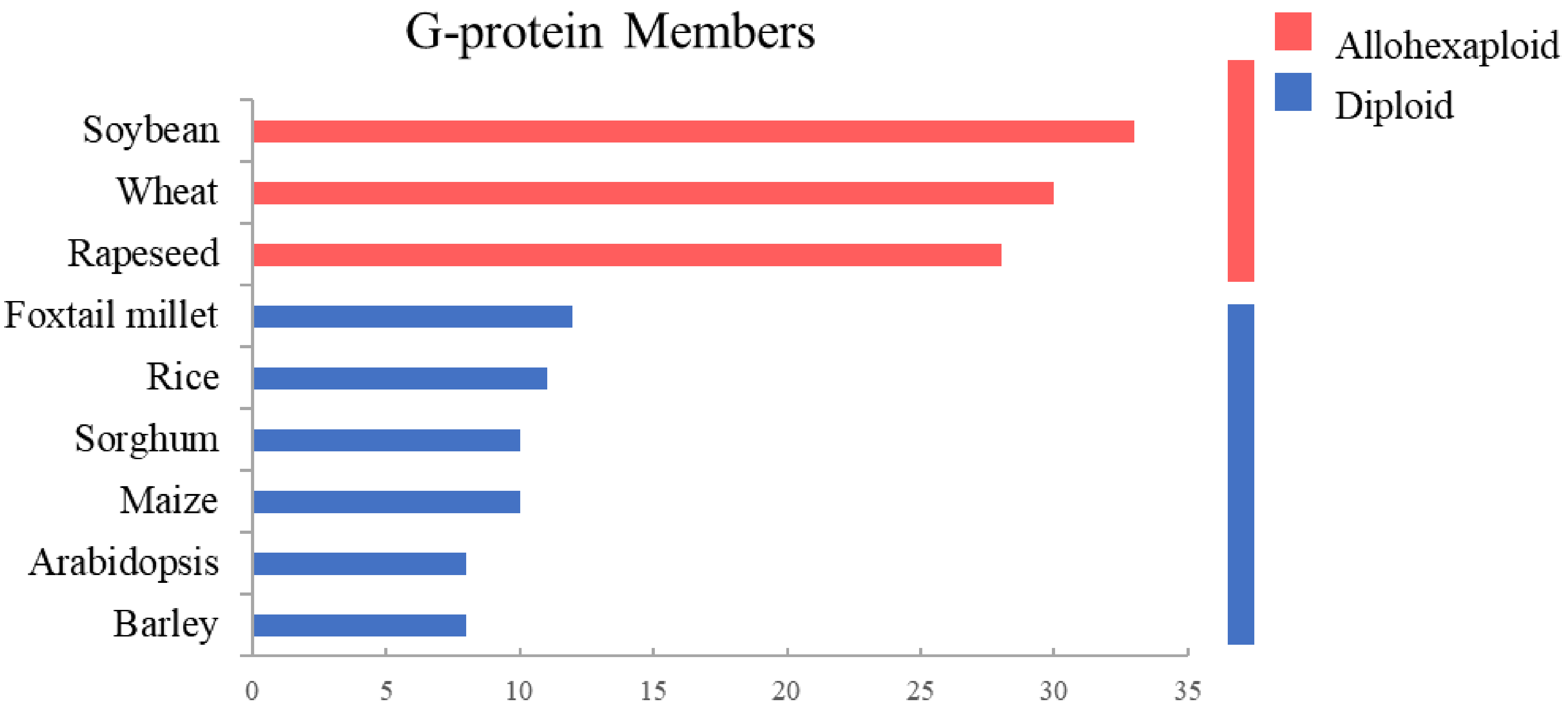

Figure S2 Phylogenetic tree of G-protein family in barley, *Arabidopsis*, and rice.

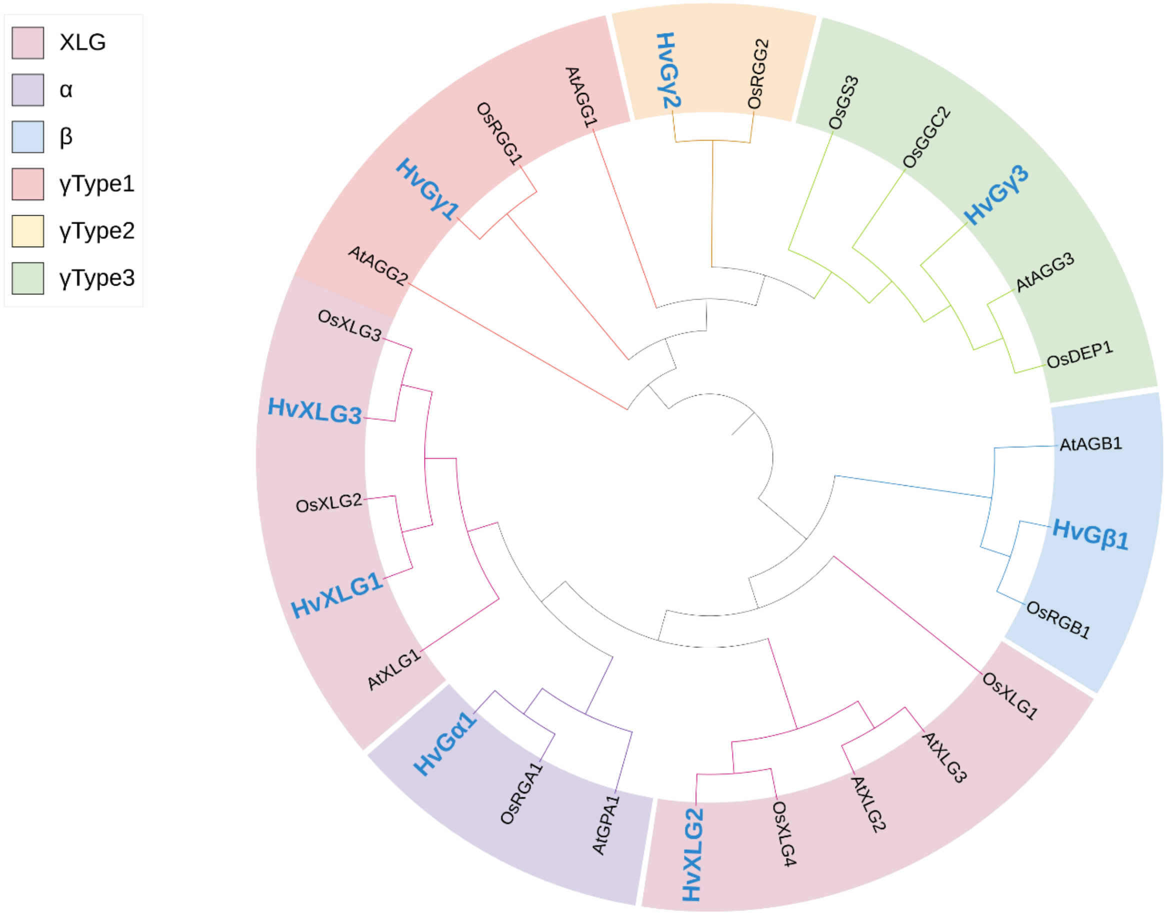

Supplement: Supplementary file 1 [file plants-13-03521-s001.zip › plants-3339444-supplementary.pdf]
